# Isavuconazole for the Treatment of Invasive Fungal Disease in Hematology Patients: A Real-World Retrospective Study on Efficacy and Safety

**DOI:** 10.3390/microorganisms13122677

**Published:** 2025-11-25

**Authors:** Pazilaiti Tuohuti, Yuhui Chen, Ailin Zhao, Jinrong Yang, He Li, Ting Niu

**Affiliations:** 1 Department of Hematology, Institute of Hematology, West China Hospital, Sichuan University, Chengdu 610041, Chinaniuting@wchscu.cn (T.N.); 2 State Key Laboratory of Biotherapy, Collaborative Innovation Center of Biotherapy, West China Hospital, Sichuan University, Chengdu 610041, China; 3 National Facility for Translational Medicine (Sichuan), West China Hospital, Sichuan University, Chengdu 610041, China; 4 West China School of Medicine, Sichuan University, Chengdu 610041, China

**Keywords:** isavuconazole, invasive fungal disease, hematology patients, prognosis

## Abstract

Invasive fungal disease (IFD) remains a life-threatening complication in patients with hematological diseases. Isavuconazole was approved by the FDA for primary treatment of invasive aspergillosis and mucormycosis. While clinical trials have demonstrated its efficacy, data on its use in hematology patients remain limited. This study aims to evaluate the real-world effectiveness and safety of isavuconazole in this population. We conducted a single-center, retrospective study of hematology patients who received isavuconazole for IFD between 1 June 2022, and 31 July 2024, at West China Hospital, Sichuan University. A total of 66 patients with proven (*n* = 9), probable (*n* = 17), or possible (*n* = 40) IFD were included in the study. Acute leukemia (AL) was the most common underlying disease, affecting 27 patients (40.9%), followed by non-Hodgkin’s lymphoma (NHL) and myelodysplastic syndrome (MDS). Over 80.0% of patients received oral isavuconazole. At 6 weeks of follow-up, a favorable response was observed in 57.6% of patients, increasing to 71.2% at 12 weeks. Factors associated with achieving complete response in isavuconazole treatment included receiving isavuconazole as primary treatment (OR = 0.10, *p* = 0.01) and reaching complete/partial remission (CR/PR) of the primary hematological disease (OR = 0.07, *p* = 0.003). The all-cause mortality rates were under 30.0%. The use of isavuconazole as primary antifungal therapy (*p* < 0.05) and achieving CR/PR in the underlying hematological disease (*p* < 0.05) were two independent predictors of improved clinical outcomes. Adverse events were reported in 33.3% of patients, and no adverse events led to discontinuation of treatment. Our study demonstrated that isavuconazole is an effective and well-tolerated treatment for IFD in hematology patients. The oral formulation provided comparable efficacy and enhanced compliance, potentially leading to improved outcomes and optimizing the management strategy. The generalizability of our findings may be limited by the single-center, retrospective nature; further validation through prospective, multi-center studies is needed.

## 1. Introduction

Invasive Fungal Disease (IFD) is a clinical syndrome resulting from the invasion of pathogenic or opportunistic fungi into the deep tissues or bloodstream of the host, leading to severe infections. It predominantly affects individuals with severely compromised immune systems [1]. Among the patients with hematological diseases, those undergoing high-intensity chemotherapy, hematopoietic stem cell transplantation (HSCT), or long-term immunosuppressive therapy were particularly at high risk for IFD [2,3]. Epidemiological studies have demonstrated that *Aspergillus* species are the most common pathogens causing IFD in patients with hematological diseases, followed by *Mucorales* [4]. The increasing resistance of these pathogens to traditional antifungal agents, coupled with the growing population of high-risk individuals, has elevated IFD to a critical challenge in the clinical management of hematological disorders. It remains a significant cause of morbidity and mortality [5,6,7,8]. Isavuconazole, a novel broad-spectrum triazole antifungal, has demonstrated favorable efficacy and safety profiles in pivotal clinical trials [9]. The international phase III SECURE trial established its non-inferiority to voriconazole for IFD treatment, with comparable day-42 all-cause mortality rates (18.6% vs. 20.2%) and a superior safety profile, with significantly fewer drug-related adverse events (42% vs. 60%; *p* < 0.001) [10]. Its favorable safety profile and dual formulation availability make it particularly suitable for immunocompromised patients. Subgroup analysis revealed that in patients with hematologic malignancies, the clinical response rate in the isavuconazole group was 15% higher than that in the control group (54% vs. 39%), suggesting its potential advantage in this population. Subsequent single-arm open-label trials further confirmed that isavuconazole achieved a 43% treatment success rate in voriconazole-resistant fungal infections [11]. Based on these results, in 2015, isavuconazole was approved by the US Food and Drug Administration (FDA) as a primary treatment for invasive aspergillosis (IA) and mucormycosis [12].

While current evidence supports the clinical use of isavuconazole, research specifically focusing on patients with hematologic diseases remains limited. Additionally, there is a paucity of clinical trials comparing the efficacy of isavuconazole in primary versus salvage therapy for IFD. This study aims to systematically evaluate the real-world efficacy and safety of isavuconazole in treating IFD among patients with hematologic diseases, thereby providing additional evidence-based insights for precision antifungal therapy.

## 2. Materials and Methods

### 2.1. Patient Population

This was a real-world, observational, retrospective study conducted at West China Hospital, Sichuan University, from 1 June 2022 to 31 July 2024. The study included patients aged 14 to 80 years with hematologic diseases who had proven, probable, or possible IFD and received isavuconazole (either orally or intravenously) for ≥7 days. Patients were excluded if they met any of the following criteria: (1) insufficient duration of isavuconazole therapy (<7 days) or non-adherence to the prescribed regimen; (2) lack of laboratory or imaging data; (3) concurrent infection with additional fungal species; (4) diagnosis of concurrent malignancies of other types; or (5) unavailability of follow-up data. Epidemiological and clinical data were collected from the Hospital Information System. The study was conducted in accordance with the Declaration of Helsinki, and the protocol was approved by the Ethics Committee of West China Hospital, Sichuan University (Approval Code: 2025-1697) on 24 September 2025. Informed consent was waived due to the retrospective design. All data were anonymized to protect patient confidentiality.

### 2.2. Definitions

IFD was diagnosed according to the criteria established by the European Organization for Research and Treatment of Cancer and the Mycoses Study Group (EORTC/MSG), and was categorized into proven, probable, and possible IFD. The proven IFD was defined as documented histopathologic and microbiological evidence of mold infection in a tissue biopsy or needle aspiration specimen from a normally sterile site, excluding BAL, cranial sinus cavity, and urine. A probable IFD was defined as the presence of at least one microbiological criterion (cytology, culture, or positive Galactomannan test results) along with one host factor (recent absolute neutrophil count (ANC) < 500 cells/mL, allogeneic stem cell transplant, T-cell immune suppressant therapy, or prolonged corticosteroid use) and one clinical criterion (nodule, cavitary, or ground-glass opacities found via computed tomography (CT); tracheobronchitis; or sinonasal infection). A possible mold infection was defined as the requirement for a host factor, along with a radiological criterion (nodules, cavitary, and ground glass opacities on CT) [13].

Initial antifungal therapy is defined as the first treatment administered following a confirmed or suspected invasive fungal infection (IFI) and continues for 7 days. Salvage antifungal therapy is defined as any treatment initiated after failure to respond to initial antifungal therapy (administered for at least 7 consecutive days). Breakthrough infection is defined as a proven, probable, or possible IFD that occurs after at least 7 days of prophylactic treatment with an antifungal agent with adequate antifungal efficacy.

Targeted treatment is defined as the use of antifungal agents specifically aimed at treating patients with microbiological evidence of *Aspergillus* or *Mucor* species, which corresponds to patients with proven or probable IFD. Empirical/diagnosis-driven treatment is defined as the administration of antifungal agents in patients with possible IFD in the absence of microbiological evidence.

The response to treatment was assessed using both clinical and radiological criteria. A complete response was defined as the complete resolution of clinical signs and symptoms, radiological abnormalities previously detected on chest X-rays or CT scans, and all relevant microbiological findings. A partial response was defined as a significant improvement (≥50%) in clinical signs and symptoms or radiological abnormalities, in the absence of persistent microbiological findings. A stable state was characterized by the absence of significant changes in clinical symptoms, signs, or radiological images. A favorable response (also termed a positive response) included both complete and partial responses. Treatment failure was defined as stable or worsening clinical or radiological findings, persistent microbiological positivity, or death. Clinical and radiological outcomes were evaluated at baseline and at 6 weeks and 12 weeks of follow-up.

Neutropenia defined as ANC ≤ 500 cells/mL at the time of diagnosis or admission. Elevated Liver Enzymes defined as a 3-fold increase in alanine aminotransferase (ALT) or aspartate aminotransferase (AST) to the baseline. All adverse events were graded according to the Common Terminology Criteria for Adverse Events (CTCAE), version 5.0.

### 2.3. Statistical Analysis

Statistical analyses were conducted using R software version 4.4.1, with data visualizations and specific statistical analyses generated in GraphPad Prism 8.0.1, and data management performed in Microsoft Excel 2021.

Continuous variables were assessed for normality using the Shapiro–Wilk test. Normally distributed data are presented as mean ± standard deviation (SD), while non-normally distributed data are reported as median (interquartile range). Group comparisons of continuous variables were performed using distribution-appropriate tests (Student’s *t*-test for normal data; Wilcoxon rank-sum test for non-normal data). Categorical variables are presented as frequencies and percentages, with between-group associations analyzed using the chi-square test or Fisher’s exact test (depending on expected cell frequencies). Baseline characteristics were summarized using the “tableone” package (version 0.13.2) in R (https://github.com/kaz-yos/tableone (accessed on 29 September 2025); https://cran.r-project.org/web/packages/tableone/index.html (accessed on 29 September 2025)). Stratified analyses of clinical responses across subgroups at 6-week and 12-week follow-up were conducted in GraphPad Prism 8.0.1, including statistical comparisons of response rates between subgroups and the generation of associated visualizations.

For identifying predictors of CR to isavuconazole, univariate logistic regression was first performed to screen variables. Variables with a *p*-value < 0.1 in the univariate analysis were included in the initial multivariable logistic regression model. A stepwise variable selection based on the Akaike Information Criterion was then applied using the “autoReg” package (version 1.0.6) in R (https://github.com/cardiomoon/autoReg (accessed on 29 September 2025); https://cardiomoon.github.io/autoReg/ (accessed on 29 September 2025)) to derive the final optimized model. The “OR (univariable)”, “OR (multivariable)”, and “OR (final)” correspond to results from these three stages, respectively.

Survival curves were analyzed using the Log-Rank test, with survival analyses and graphing performed in GraphPad Prism 8.0.1. All tests were two-sided, and a *p*-value < 0.05 was considered statistically significant.

## 3. Results

### 3.1. Demographics and Clinical Characteristics

A total of 66 patients with hematologic diseases who received oral or intravenous isavuconazole for the treatment of IFD between 1 June 2022, and 31 July 2024, were enrolled in this study. The median age of the patients was 52 years, and 60.6% (*n* = 40) were male. Acute leukemia (AL) was the most common underlying disease, affecting 27 patients (40.9%), followed by non-Hodgkin’s lymphoma (NHL) and myelodysplastic syndrome (MDS). Aplastic anemia, the only benign disease included, was present in 9% of the patients (Table 1). Among the cohort, 37 patients (56.1%) had undergone chemotherapy, and 25 patients (37.9%) had received HSCT. At the time of IFD diagnosis, nearly half of the patients (*n* = 32, 48.5%) had achieved complete or partial remission in their underlying hematological disease. Neutropenia was present in 16 patients (24.2%) at the onset of IFDs. Concurrent bacterial infections were observed in 26 patients (39.4%), while viral infections were present in 24 patients (36.4%) (Table 1). Over 60% of the patients had no history of previous fungal infections (Table 2).

### 3.2. Invasive Fungal Disease

As previously defined, IFD was categorized into proven (9/66, 13.6%), probable (17/66, 25.8%), and possible IFD (40/66, 60.6%). Fungal culture results were positive in only 24 patients (36.4%), with the most common pathogens being *Aspergillus* (11/24, 45.8%). The most common site of IFD involvement was the lung (63/66, 95.5%). Targeted treatment was administered to 20 patients, while the remaining 46 patients received empirical/diagnosis-driven treatment in the absence of microbiological evidence (Table 1).

Among the cohort, 28 patients (42.4%) received antifungal prophylaxis prior to the onset of IFD. The antifungal regimens predominantly included triazole antifungals (*n* = 19, 67.9%) and echinocandins (*n* = 5, 17.9%) (Table 2).

Isavuconazole was utilized as salvage therapy in 42 cases (63.6%) and as primary therapy in 24 cases (36.4%). The two groups were largely comparable in most clinical characteristics. However, significant differences were observed between the groups with respect to age, underlying hematological disease, status of hematological disease, fungal culture results (Table 1), history of previous fungal infection, and prior receipt of antifungal prophylaxis therapy (Table 2).

Among the 42 patients who received isavuconazole as salvage therapy, the initial antifungal regimens were predominantly composed of other triazole antifungals (*n* = 17, 40.5%; including voriconazole and posaconazole). Notably, over 60% of the cohort had prior exposure to triazole antifungals before initiating isavuconazole salvage therapy. The main reasons for switching to isavuconazole included refractoriness to primary antifungal agents (*n* = 24, 57.1%) and intolerance to initial regimens (*n* = 15, 35.7%).

In this study, isavuconazole capsule was the most common dosage form. Over 80% of patients received isavuconazole as monotherapy via the oral route. Additionally, 28 patients underwent combined antifungal therapy, which included isavuconazole in combination with echinocandins (*n* = 11, 39.3%) or polyenes (*n* = 17, 60.7%) (Table 2).

### 3.3. Outcomes

Overall, 48 patients (72.7%) achieved a favorable response, with 36 of them achieving a complete response (Table 2). The complete response rate among patients who received isavuconazole for primary treatment was 70.8% (17/24), which was significantly higher than the rate of 45.2% (19/42) observed in patients who received isavuconazole for salvage treatment. According to univariate analysis, complete response was significantly higher among patients who received isavuconazole for primary treatment compared to those who received it for salvage treatment (17/24, 70.8% vs. 19/42, 45.2%; odds ratio [OR] = 0.34, *p* = 0.048; Table 3). Additionally, complete response was higher among patients who had achieved complete or partial remission (CR/PR) in their underlying hematological diseases compared to those who remained in non-remission or untreated (22/32, 68.8% vs. 14/34, 41.2%; OR = 0.32, *p* = 0.027; Table 3). Complete response also was higher in non-neutropenic patients compared to patients who had neutropenia at the onset of IFDs (31/50, 62.0% vs. 5/16, 31.3%; OR = 0.28, *p* = 0.037; Table 3).

Multivariate analysis shows that receiving isavuconazole for primary treatment (OR = 0.10, *p* = 0.013) and achieving complete or partial remission in the underlying hematological disease (OR = 0.07, *p* = 0.002) were significant independent predictors of achieving CR during isavuconazole treatment (Table 3).

**Figure 1:**
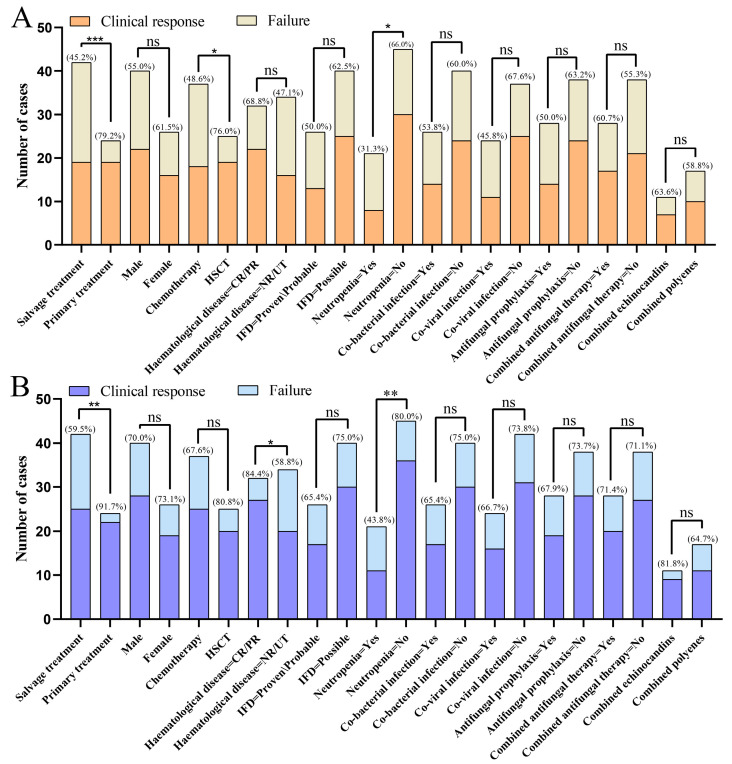
Clinical responses in different subgroups at 6-week (**A**) and 12-week (**B**) follow up. Abbreviations: IFD, Invasive fungal disease; HSCT, Hematopoietic stem cell transplantation; CR, Complete response; PR, Partial response; NR, Non-response; UT, Untreated; *, *p*-value < 0.05; **, *p*-value < 0.01; ***, *p*-value < 0.001; ns, not significant.

Within the 6-week follow-up period, a cumulative favorable response was achieved in 38 patients (57.6%, Table 2). Figure 1A presents a stratified analysis of clinical outcomes across subgroups at the 6-week endpoint. Notably, patients receiving isavuconazole as primary antifungal therapy demonstrated significantly higher response rates compared to those in the salvage therapy cohort (79.2% vs. 45.2%; *p* = 0.0097). Furthermore, enhanced efficacy was observed in HSCT recipients (76.0% vs. 48.6% in patients receiving chemotherapy, *p* = 0.0313), as well as in non-neutropenic patients (66.0% vs. 31.2% in neutropenic counterparts, *p* = 0.0204).

After an additional 6-week follow-up, over 70% of patients achieved a favorable response (Table 2). The favorable response rate remained significantly higher among patients receiving isavuconazole as primary treatment compared to the salvage treatment subgroup (91.7% vs. 59.5%, *p* = 0.0054; Figure 1B). Higher response rates were observed in patients who had achieved CR or PR in their underlying hematological disease (84.4% vs. 58.8% in non-remission/untreated [NR/UT] patients, *p* = 0.0301; Figure 1B) and in non-neutropenic patients (80.0% vs. 43.8% in neutropenic patients, *p* = 0.0090; Figure 1B). Compared to the first 6-week follow-up, the difference in response rates between the two treatment groups for underlying hematological disease was no longer statistically significant (80.0% vs. 67.6%, *p* = 0.387; Figure 1B).

**Figure 2:**
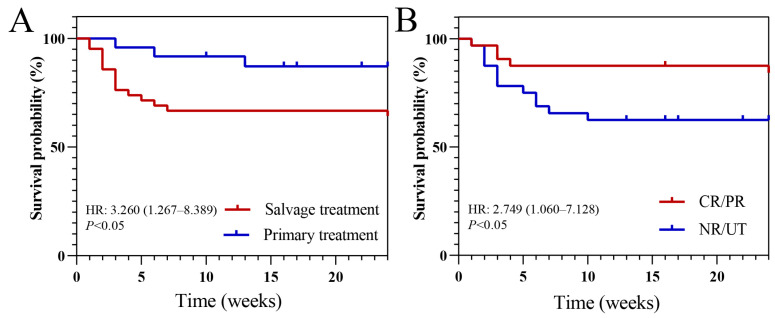
Survival analysis of different subgroups. (**A**) Survival analysis of using isavuconazole as primary treatment and salvage treatment; (**B**) Survival analysis of achieving complete or partial remission (CR/PR) in the primary hematologic disease and remained non-remission and un-treated (NR/UT). HR, hazard ratio.

Among the entire cohort, the all-cause mortality rates were 27.3% and 28.8% at 6 weeks and 12 weeks of follow-up, respectively. The all-cause mortality rate in the primary treatment group was 16.7% (4/24), which was lower than the 35.7% (15/42) observed in the salvage treatment group (Table 2). Survival analysis revealed that receiving isavuconazole as primary antifungal therapy (hazard ratio [HR]: 3.260, *p* < 0.05; Figure 2A) and achieving CR or PR in the primary hematologic disease (HR: 2.749, *p* < 0.05; Figure 2B) were independently associated with improved survival probabilities.

### 3.4. Treatment Related Adverse Events

Adverse events that were possibly related to isavuconazole were reported in 22 patients (33.3%). The most common adverse event was elevated liver enzymes, which was observed in 17 patients, all cases were grade 1–2 in severity. One patient in the salvage treatment group developed grade 3 thrombocytopenia, which resolved following blood transfusion and medication. Nausea/vomiting and back pain, considered potentially treatment-related, were reported in three and one patient(s), respectively. (Table 4). All adverse events did not lead to isavuconazole dose adjustment and resolved with supportive medication. No adverse events led to permanent discontinuation of isavuconazole therapy.

## 4. Discussion

In this study, we report real-world clinical data from patients with diverse hematological diseases who were treated with either oral or intravenous isavuconazole for IFD. The primary aim of this investigation was to assess the antifungal efficacy and safety profile of isavuconazole in this high-risk patient population.

The respiratory tract was the most common site of IFD, with *Aspergillus* spp. being the predominant pathogen isolated. Acute leukemia represented the most frequent underlying hematological disease, a finding consistent with prior reports [4,5]. The predominance of pulmonary involvement aligns with the inhalation route of *Aspergillus* conidia [14].

The overall clinical response rate was 73%, which compares favorably with the rates reported in the SECURE trial (62%) and the VITAL trial (58%) [10,11]. Notably, isavuconazole monotherapy demonstrated a response rate of 71.1%, significantly higher than those observed in previous studies—including the 35% response for isavuconazole and 36% for voriconazole in the SECURE trial, as well as historical rates of 52.8% for voriconazole monotherapy and 31.6% for amphotericin B monotherapy [15]. Furthermore, the all-cause 12-week mortality rate in our cohort (28.8%) was comparable to that of the isavuconazole arms in both the SECURE (29%) and VITAL (34%) trials, as well as to previously reported mortality rates for posaconazole and amphotericin B [16,17].

Our study demonstrated a significantly higher complete response rate in patients receiving isavuconazole as primary therapy compared to salvage therapy (70.8% vs. 45.2%). This trend was consistently observed for clinical response rates at both 6-week and 12-week follow-up intervals, suggesting that earlier initiation of isavuconazole therapy is positively associated with improved treatment outcomes. The superior outcomes observed in the primary therapy group may be attributed to a confluence of pharmacological and clinical advantages. First, the broad antifungal spectrum of isavuconazole, encompassing both *Aspergillus* and *Mucorales* [18], allows for early and effective coverage, potentially curbing initial fungal progression, minimizing tissue invasion (e.g., angioinvasion), and preventing dissemination—a critical factor for improving prognosis. Second, its favorable safety profile, characterized by lower incidences of hepatotoxicity and nephrotoxicity compared to alternatives like voriconazole or amphotericin B, enhances treatment continuity. This results in fewer therapy disruptions, enabling patients to maintain consistent drug exposure and achieve therapeutic blood levels more reliably, which is paramount for clinical efficacy. However, as a retrospective analysis, these findings should be interpreted with caution. Finally, the use of the oral formulation often selects for patients with a more stable baseline condition who can tolerate oral intake. While this introduces a selection bias, it underscores that isavuconazole is a viable and effective option for initiating treatment in such patients, facilitating early intervention.

In the SECURE trial, >75% of the 258 patients receiving isavuconazole initiated therapy with intravenous formulation before transitioning to capsule [10]. In contrast, our study demonstrated that >80% of patients received isavuconazole capsules as initial therapy. Notably, our observed clinical response rates exceeded those reported in the SECURE trial, supporting the therapeutic efficacy of the isavuconazole capsule. The oral formulation maintains comparable clinical effectiveness while offering distinct advantages in treatment convenience and outpatient management, potentially enhancing patient compliance.

Unlike previous trials, our study specifically included patients who had received first-line treatment with other mold-active azoles (voriconazole or posaconazole), representing >60% of the salvage therapy cohort. These findings further validate isavuconazole’s effectiveness as salvage therapy following failure of other azoles.

The present study demonstrates that during the initial 6-week follow-up period, HSCT recipients exhibited superior clinical response rates to isavuconazole treatment compared to patients receiving chemotherapy alone. While this difference became statistically non-significant after 12 weeks of follow-up, HSCT patients maintained a numerically higher response rate (80.0% vs. 67.6%), suggesting potentially greater clinical efficacy of isavuconazole in the transplant population subgroup, and survival analyses further revealed that patients who had a CR or PR of their underlying hematological disease showed better treatment outcomes with isavuconazole at both 6-week and 12-week follow-up intervals compared to non-responders or untreated patients. This improved outcome may be attributed to several factors: (1) the restoration of cellular immunity in patients achieving CR/PR of their underlying hematological diseases and (2) reduced requirement for immunosuppressive agents during IFD treatment compared to NR/UT patients.

Previous studies have demonstrated that systemic antifungal agents are associated with a broad spectrum of adverse events, primarily including hepatotoxicity, nephrotoxicity, cardiovascular toxicity, and neurotoxicity, as well as gastrointestinal and cutaneous reactions [6]. In the SECURE trial, drug-related adverse events were reported in 42% of isavuconazole-treated patients versus 60% receiving voriconazole [10]. A phase 3 randomized study by Maertens et al. reported treatment-related adverse event rates of 30% for posaconazole compared to 40% for voriconazole [19]. Batista et al. observed overall treatment-related adverse events in 21% of patients receiving primary antifungal therapy, with incidence rates of 3% for isavuconazole, 34% for voriconazole, and 23% for amphotericin B [20]. Consistent with these findings, our study documented an overall adverse event rate of 33% with isavuconazole, further supporting its advantageous safety profile relative to other antifungal agents.

Notably, over 60% of patients in this study were classified as possible IFD. This can be largely attributed to the fact that many hematology patients, due to conditions such as severe anemia, thrombocytopenia, and coagulopathy, are often ineligible for invasive procedures like biopsies or aspirates, precluding the acquisition of definitive histopathological or microbiological evidence, which is essential for a proven/probable IFD diagnosis. Consequently, diagnosis in most of these cases had to rely on clinical symptoms and radiological findings, leading to the high proportion of possible IFD. This distribution, however, is consistent with the clinical reality in the hematologic patient population, underscoring the diagnostic challenges for IFD in hematologic patients—particularly those at high risk from invasive procedures. As the diagnosis in these cases primarily relies on clinical manifestations, host factors, and radiological changes, there is an inherent possibility of misclassifying some non-IFD cases as possible IFD. However, thorough differential diagnoses were clinically performed to exclude alternative explanations, such as those attributable to the underlying hematological disease or bacterial/viral infections, for the observed clinical and radiographic presentations. Therefore, it is considered that the high proportion of possible IFD is unlikely to substantially impact the treatment efficacy and clinical outcomes observed in this study.

This study has several limitations that should be considered when interpreting the results. First, in this real-world study, some baseline characteristics were unevenly distributed between groups. While this reflects the clinical heterogeneity of the patient population, it introduces a potential for confounding that could not be fully addressed with our sample size through advanced statistical methods. Therefore, the association between treatment and outcomes should be considered exploratory and necessitates verification in larger, prospective studies. Second, as a single-center study, our patient population was derived from a specific geographical context. The patient demographics might not be fully representative of those in other regions. Therefore, further validation through prospective, multi-center studies is needed to confirm our conclusions.

## 5. Conclusions

This study demonstrates that isavuconazole represents an effective therapeutic option for IFD in patients with hematological diseases, demonstrating high clinical response rates in both primary and salvage treatment settings. Our findings indicate superior outcomes when isavuconazole is administered as primary therapy and in patients achieving complete or partial remission of their underlying hematological disease. Notably, isavuconazole exhibited a more favorable safety profile compared to historical data on other antifungal agents, with significantly lower toxicity rates. These results, combined with the demonstrated efficacy of oral administration (which may improve treatment compliance while maintaining clinical effectiveness), position isavuconazole as a particularly promising therapeutic choice for IFD management in this high-risk patient population.

## Figures and Tables

**Table 1:** Baseline Characteristics.

Characteristic	Overall, N = 66	Salvage Treatment, N = 42	Primary Treatment, N = 24	*p*-Value
Age (years), (median [IQR])	52.00 [41.00, 58.75]	49.00 [34.75, 55.50]	55.50 [48.75, 61.50]	0.046
Sex (%)				
Male	40/66 (60.6)	25/42 (59.5)	15/24 (62.5)	1
Female	26/66 (39.4)	17/42 (40.5)	9/24 (37.5)	
Primary disease (%)				0.009
AA	6/66 (9.1)	5/42 (11.9)	1/24 (4.2)	
AL	27/66 (40.9)	24/42 (57.1)	3/24 (12.5)	
CL	2/66 (3.0)	0/42 (0.0)	2/24 (8.3)	
HL	2/66 (3.0)	1/42 (2.4)	1/24 (4.2)	
MDS	8/66 (12.1)	4/42 (9.5)	4/24 (16.7)	
MM	5/66 (7.6)	2/42 (4.8)	3/24 (12.5)	
NHL	10/66 (15.2)	4/42 (9.5)	6/24 (25.0)	
Others	6/66 (9.1)	2/42 (4.8)	4/24 (16.7)	
Treatment for underlying hematological diseases (%)				0.096
Chemotherapy	37/66 (56.1)	20/42 (47.6)	17/24 (70.8)	
HSCT	25/66 (37.9)	20/42 (47.6)	5/24 (20.8)	
Untreated	4/66 (6.1)	2/42 (4.8)	2/24 (8.3)	
Status of hematological disease at IFD (%)				0.002
CR/PR	32/66 (48.5)	27/42 (64.3)	5/24 (20.8)	
NR	29/66 (43.9)	11/42 (26.2)	18/24 (75.0)	
UT	3/66 (4.5)	2/42 (4.8)	1/24 (4.2)	
UK	2/66 (3.0)	2/42 (4.8)	0/24 (0.0)	
Classifications IFD (%)				0.127
Proven	9/66 (13.6)	8/42 (19.0)	1/24 (4.2)	
Probable	17/66 (25.8)	12/42 (28.6)	5/24 (20.8)	
Possible	40/66 (60.6)	22/42 (52.4)	18/24 (75.0)	
Positive fungal culture (%)	24/66 (36.4)	20/42 (47.6)	4/24 (16.7)	0.025
Species identified (%)				
*Aspergillus*	11/66 (16.7)	10/42 (23.8)	1/24 (4.2)	
*Candida*	4/66 (6.1)	1/42 (2.4)	3/24 (12.5)	
*Fusarium*	1/66 (1.5)	1/42 (2.4)	0/24 (0.0)	
*Mucor*	6/66 (9.1)	5/42 (11.9)	1/24 (4.2)	
*Pneumocystis*	1/66 (1.5)	1/42 (2.4)	0/24 (0.0)	
*Rhizopus*	3/66 (4.5)	2/42 (4.8)	1/24 (4.2)	
*Yeast*	1/66 (1.5)	1/42 (2.4)	0/24 (0.0)	
Null	39/66 (59.1)	21/42 (50.0)	18/24 (75.0)	
Site of IFD (%)				0.408
CNS	1/66 (1.5)	1/42 (2.4)	0/24 (0.0)	
Lung	63/66 (95.5)	40/42 (95.2)	23/24 (95.8)	
Lung + CNS	1/66 (1.5)	1/42 (2.4)	0/24 (0.0)	
Maxillofacial	1/66 (1.5)	0/42 (0.0)	1/24 (4.2)	
Neutropenia (%)	16/66 (24.2)	12/42 (28.6)	4/24 (16.7)	0.431
Duration of neutropenia (%)				0.209
≤7 d	6/66 (9.1)	6/42 (14.3)	0/24 (0.0)	
7–14 d	1/66 (1.5)	1/42 (2.4)	0/24 (0.0)	
>14 d	9/66 (13.6)	5/42 (11.9)	4/24 (16.7)	
Null	50/66 (75.8)	30/42 (71.4)	20/24 (83.3)	
Combined bacterial infections (%)	26/66 (39.4)	15/42 (35.7)	11/24 (45.8)	0.584
Combined viral infection (%)	24/66 (36.4)	18/42 (42.9)	6/24 (25.0)	0.236
Laboratory findings				
WBC (×10^9^/L), (median [IQR])	2.92 [1.26, 5.47]	2.83 [0.88, 5.89]	3.33 [1.64, 4.04]	0.709
NEUT (×10^9^/L), (median [IQR])	2.24 [0.50, 4.38]	2.28 [0.18, 4.54]	1.94 [0.75, 2.68]	0.668
PLT (×10^9^/L), (median [IQR])	28.00 [11.00, 116.00]	17.00 [7.00, 73.00]	81.50 [23.25, 164.50]	0.013
HB (g/L), (median [IQR])	75.00 [64.00, 99.00]	76.00 [64.00, 99.00]	72.50 [62.50, 91.75]	0.946
ALT (IU/L), (median [IQR])	22.00 [11.00, 42.00]	22.00 [12.00, 44.25]	22.00 [10.00, 28.05]	0.479
AST (IU/L), (median [IQR])	21.00 [15.00, 32.00]	20.00 [15.00, 34.50]	23.00 [13.00, 26.00]	0.544
TBIL (umol/L), (median [IQR])	9.00 [6.55, 13.70]	9.70 [7.75, 13.95]	7.90 [6.15, 12.75]	0.328
DBIL (umol/L), (median [IQR])	3.70 [2.30, 6.60]	4.25 [2.48, 7.03]	2.90 [1.95, 5.65]	0.139
IBIL (umol/L), (median [IQR])	5.90 [3.90, 8.60]	6.05 [4.25, 8.53]	5.60 [3.75, 9.05]	0.534
Scr (umol/L), (median [IQR])	70.00 [53.00, 101.75]	84.00 [53.00, 107.00]	61.00 [53.00, 77.00]	0.284
BUN (mmol/L), (median [IQR])	6.20 [4.32, 8.50]	6.80 [4.90, 9.95]	5.50 [4.15, 7.65]	0.143

Abbreviations: AA, Acute myeloid leukemia; AL, Acute lymphoblastic leukemia; CL, Chronic lymphocytic leukemia; HL, Hodgkin’s lymphoma; MDS, Myelodysplastic syndrome; MM, Multiple myeloma; NHL, Non-Hodgkin’s lymphoma; IFD, Invasive fungal disease; CNS, Central nervous system; HSCT, Hematopoietic stem cell transplantation; CR, Complete response; PR, Partial response; NR, Non-response; UK, Unknown; UT, Untreated; WBC, White blood cell count; NEUT, Neutrophil count; PLT, Platelet count; Hb, Hemoglobin; ALT, Alanine aminotransferase; AST, Aspartate aminotransferase; TBIL, Total bilirubin; DBIL, Direct bilirubin; IBIL, Indirect bilirubin; Scr, Serum creatinine; BUN, Blood urea nitrogen; IQR, Interquartile range.

**Table 2:** Characteristics related to antifungal treatment and outcomes.

Characteristic	Overall, N = 66	Salvage Treatment, N = 42	Primary Treatment, N = 24	*p*-Value
History of previous fungal infection (%)				0.049
Yes	19/66 (28.8)	14/42 (33.3)	5/24 (20.8)	
No	41/66 (62.1)	22/42 (52.4)	19/24 (79.2)	
UK	6/66 (9.1)	6/42 (14.3)	0/24 (0.0)	
Antifungal prophylaxis (%)				0.009
Yes	28/66 (42.4)	23/42 (54.8)	5/24 (20.8)	
No	36/66 (54.5)	17/42 (40.5)	19/24 (79.2)	
UK	2/66 (3.0)	2/42 (4.8)	0/24 (0.0)	
Type of prophylaxis (%)				0.098
Echinocandins	5/66 (7.6)	4/42 (9.5)	1/24 (4.2)	
Other Azoles	19/66 (28.8)	15/42 (35.7)	4/24 (16.7)	
Echinocandins + Other Azoles	2/66 (3.0)	2/42 (4.8)	0/24 (0.0)	
Echinocandins + Polyenes	2/66 (3.0)	2/42 (4.8)	0/24 (0.0)	
Null	38/66 (57.6)	19/42 (45.2)	19/24 (79.2)	
Contains Other Azoles (%)	26/66 (39.4)	26/42 (61.9)		
Fungal treatment prior to Isavuconazole (%)				
Echinocandins		1/42 (2.4)		
Other Azoles		17/42 (40.5)		
Polyenes		8/42 (19.0)		
Other Azoles + Echinocandins		1/42 (2.4)		
Polyenes + Echinocandins		7/42 (16.7)		
Polyenes + Other Azoles		8/42 (19.0)		
Duration of fungal treatment before Isavuconazole (median [IQR])		8.00 [5.00, 20.00]		
Reason for switching to Isavuconazole (%)				
Ineffectiveness		24/42 (57.1)		
Intolerance		15/42 (35.7)		
Poor compliance		1/42 (2.4)		
Sequential therapy		2/42 (4.8)		
Therapy (%)				0.123
Target treatment	20/66 (30.3)	16/42 (38.1)	4/24 (16.7)	
Empirical/Diagnosis-driven treatment	44/66 (69.7)	26/42 (61.9)	20/24 (83.3)	
Combined antifungal therapy (%)				0.687
Echinocandins	11/66 (16.7)	6/42 (14.3)	5/24 (20.8)	
Polyenes	17/66 (25.8)	12/42 (28.6)	5/24 (20.8)	
Null	38/66 (57.6)	24/42 (57.1)	14/24 (58.3)	
Dosage form (%)				0.051
Intravenous administration	8/66 (12.1)	7/42 (16.7)	1/24 (4.2)	
Oral	53/66 (80.3)	30/42 (71.4)	23/24 (95.8)	
Oral + Intravenous administration	5/66 (7.6)	5/42 (11.9)	0/24 (0.0)	
Response to Isavuconazole therapy (%)				0.136
CR	36/66 (54.5)	19/42 (45.2)	17/24 (70.8)	
PR	12/66 (18.2)	7/42 (16.7)	5/24 (20.8)	
SD	9/66 (13.6)	8/42 (19.0)	1/24 (4.2)	
PD	1/66 (1.5)	1/42 (2.4)	0/24 (0.0)	
UK	8/66 (12.1)	7/42 (16.7)	1/24 (4.2)	
6th week response (%)				0.02
Positive	38/66 (57.6)	19/42 (45.2)	19/24 (79.2)	
Negative	17/66 (25.8)	13/42 (31.0)	4/24 (16.7)	
UK	11/66 (16.7)	10/42 (23.8)	1/24 (4.2)	
12th week response (%)				0.021
Positive	47/66 (71.2)	25/42 (59.5)	22/24 (91.7)	
Negative	11/66 (16.7)	10/42 (23.8)	1/24 (4.2)	
UK	8/66 (12.1)	7/42 (16.7)	1/24 (4.2)	
6th week death (%)	18/66 (27.3)	14/42 (33.3)	4/24 (16.7)	0.24
12th week death (%)	19/66 (28.8)	15/42 (35.7)	4/24 (16.7)	0.173

Abbreviations: CR Complete response; PR Partial response; SD, Stable disease; PD, Progressive disease; UK, Unknown.

**Table 3:** Univariate and Multivariate Analysis of Factors Influencing CR.

Name		CR (N = 36)	PR/SD/PD (N = 30)	OR (Univariable)	OR (Multivariable)	OR (Final)
The purpose of using Isavuconazole	Primary treatment	17/36 (47.2%)	7/30 (23.3%)			
	Salvage treatment	19/36 (52.8%)	23/30 (76.7%)	0.34 (0.12–0.99, *p* = 0.048)	0.10 (0.02–0.61, *p* = 0.013)	0.10 (0.02–0.56, *p* = 0.008)
Sex	Male	20/36 (55.6%)	20/30 (66.7%)			
	Female	16/36 (44.4%)	10/30 (33.3%)	1.60 (0.59–4.37, *p* = 0.359)		
Treatment	Non-HSCT	21/36 (58.3%)	20/30 (66.7%)			
	HSCT	15/36 (41.7%)	10/30 (33.3%)	1.43 (0.52–3.91, *p* = 0.488)		
Status of hematological disease at IFD	CR/PR	22/36 (61.1%)	10/30 (33.3%)			
	NR/UT	14/36 (38.9%)	20/30 (66.7%)	0.32 (0.12–0.88, *p* = 0.027)	0.06 (0.01–0.44, *p* = 0.005)	0.07 (0.01–0.36, *p* = 0.002)
Classifications IFD	Proven\Probable	11/36 (30.6%)	15/30 (50%)			
	Possible	25/36 (69.4%)	15/30 (50%)	2.27 (0.83–6.22, *p* = 0.110)	1.12 (0.23–5.47, *p* = 0.893)	
Positive fungal culture	No	27/36 (75%)	15/30 (50%)			
	Yes	9/36 (25%)	15/30 (50%)	0.33 (0.12–0.94, *p* = 0.038)	0.33 (0.06–1.80, *p* = 0.201)	0.31 (0.08–1.14, *p* = 0.078)
Neutropenia	No	31/36 (86.1%)	19/30 (63.3%)			
	Yes	5/36 (13.9%)	11/30 (36.7%)	0.28 (0.08–0.93, *p* = 0.037)	1.10 (0.21–5.74, *p* = 0.906)	
Combined bacterial infections	No	24/36 (66.7%)	16/30 (53.3%)			
	Yes	12/36 (33.3%)	14/30 (46.7%)	0.57 (0.21–1.55, *p* = 0.271)		
Combined viral infection	No	24/36 (66.7%)	18/30 (60%)			
	Yes	12/36 (33.3%)	12/30 (40%)	0.75 (0.27–2.05, *p* = 0.576)		
Antifungal prophylaxis	No	22/36 (61.1%)	16/30 (53.3%)			
	Yes	14/36 (38.9%)	14/30 (46.7%)	0.73 (0.27–1.94, *p* = 0.525)		
Combined antifungal therapy	No	19/36 (52.8%)	19/30 (63.3%)			
	Yes	17/36 (47.2%)	11/30 (36.7%)	1.55 (0.57–4.16, *p* = 0.389)		
Age, years (Mean ± SD)		48.4 ± 15.3	49.4 ± 17.0	1.00 (0.97–1.03, *p* = 0.797)		

Abbreviations: IFD, Invasive fungal disease; HSCT, Hematopoietic stem cell transplantation; CR, Complete response; PR, Partial response; NR, Non-response; UT, Untreated; SD, Standard deviation; OR, Odds ratio.

**Table 4:** Adverse Events Related to Isavuconazole.

AE Type	Salvage Treatment, n (%)	Primary Treatment, n (%)
Elevated Liver Enzymes ^1^		
Grade 1–2	12/42 (28.6%)	5/24 (20.8%)
Grade 3–4	0/42 (0.0%)	0/24 (0.0%)
Nausea/Vomiting		
Grade 1–2	3/42 (7.1%)	0/24 (0.0%)
Grade 3–4	0/42 (0.0%)	0/42 (0.0%)
Thrombocytopenia		
Grade 1–2	0/42 (0.0%)	0/24 (0.0%)
Grade 3–4	1/42 (2.4%)	0/24 (0.0%)
Back Pain	0/42 (0.0%)	1/24 (4.2%)

^1^ Elevated liver enzymes defined as a three-fold increase in alanine aminotransferase (ALT) or aspartate aminotransferase (AST) to the baseline. Abbreviations: AE, Adverse Events.

## Data Availability

The original contributions presented in this study are included in the article/supplementary material. Further inquiries can be directed to the corresponding author.

## Supplementary Materials

The following supporting information can be downloaded at: https://www.mdpi.com/article/10.3390/microorganisms13122677/s1, raw data.

## Abbreviations

The following abbreviations are used in this manuscript:

IFD	Invasive fungal disease
IFI	Invasive fungal infection
HSCT	Hematopoietic stem cell transplantation
FDA	Food and Drug Administration
IA	Invasive aspergillosis
ANC	Absolute neutrophil count
ALT	Alanine aminotransferase
AST	Aspartate aminotransferase
AA	Aplastic anemia
AL	Acute leukemia
CL	Chronic leukemia
HL	Hodgkin’s lymphoma
MDS	Myelodysplastic syndrome
MM	Multiple myeloma
NHL	Non-Hodgkin’s lymphoma
CR	Complete remission
PR	Partial remission
SD	Stable disease
PD	Progressive disease
